# Filtering-induced changes of pulse transmit time across different ages: a neglected concern in photoplethysmography-based cuffless blood pressure measurement

**DOI:** 10.3389/fphys.2023.1172150

**Published:** 2023-07-25

**Authors:** Shangdi Liao, Haipeng Liu, Wan-Hua Lin, Dingchang Zheng, Fei Chen

**Affiliations:** ^1^ Department of Electronic and Electrical Engineering, Southern University of Science and Technology, Shenzhen, China; ^2^ Research Centre for Intelligent Healthcare, Coventry University, Coventry, United Kingdom; ^3^ Chinese Academy of Sciences Key Laboratory of Human-Machine Intelligence-Synergy Systems, Shenzhen Institute of Advanced Technology, Shenzhen, China

**Keywords:** cuffless blood pressure measurement, pulse transit time (PTT), photoplethysmography (PPG), filtering, waveform feature

## Abstract

**Background:** Pulse transit time (PTT) is a key parameter in cuffless blood pressure measurement based on photoplethysmography (PPG) signals. In wearable PPG sensors, raw PPG signals are filtered, which can change the timing of PPG waveform feature points, leading to inaccurate PTT estimation. There is a lack of comprehensive investigation of filtering-induced PTT changes in subjects with different ages.

**Objective:** This study aimed to quantitatively investigate the effects of aging and PTT definition on the infinite impulse response (IIR) filtering-induced PTT changes.

**Methods:** One hundred healthy subjects in five different ranges of age (i.e., 20–29, 30–39, 40–49, 50–59, and over 60 years old, 20 subjects in each) were recruited. Electrocardiogram (ECG) and PPG signals were recorded simultaneously for 120 s. PTT was calculated from the R wave of ECG and PPG waveform features. Eight PTT definitions were developed from different PPG waveform feature points. The raw PPG signals were preprocessed then further low-pass filtered. The difference between PTTs derived from preprocessed and filtered PPG signals, and the relative difference, were calculated and compared among five age groups and eight PTT definitions using the analysis of variance (ANOVA) or Scheirer–Ray–Hare test with *post hoc* analysis. Linear regression analysis was used to investigate the relationship between age and filtering-induced PTT changes.

**Results:** Filtering-induced PTT difference and the relative difference were significantly influenced by age and PTT definition (*p* < 0.001 for both). Aging effect on filtering-induced PTT changes was consecutive with a monotonous trend under all PTT definitions. The age groups with maximum and minimum filtering-induced PTT changes depended on the definition. In all subjects, the PTT defined by maximum peak of PPG had the minimum filtering-induced PTT changes (mean: 16.16 ms and 5.65% for PTT difference and relative difference). The changes of PTT defined by maximum first PPG derivative had the strongest linear relationship with age (R-squared: 0.47 and 0.46 for PTT difference relative difference).

**Conclusion:** The filtering-induced PTT changes are significantly influenced by age and PTT definition. These factors deserve further consideration to improve the accuracy of PPG-based cuffless blood pressure measurement using wearable sensors.

## 1 Introduction

Photoplethysmography (PPG) signal reflects the volumetric changes in microcirculation. PPG pulse waveform characteristics contain vital information regarding cardiovascular systems and associated diseases. The PPG technology has been widely used in physiological measurement of important cardiovascular parameters, e.g., heart rate, heart rate variability, and blood pressure (Allen, 2007; Liu et al., 2019; Khalid et al., 2020; Khalid et al., 2022). Nowadays, the development of wearable technology further expanded the application scenarios of PPG-based mobile health monitoring in daily life.

Among many, pulse transit time (PTT) is one of the most important characteristics provided by PPG pulse waveform. PTT refers the time for heart pulse wave (PW) to propagate through a length of the arterial tree. It can be approximated as the interval between the R wave of electrocardiogram (ECG) and the characteristic point of PPG signal (e.g., the end-of-diastolic valley) in the same cardiac cycle. PTT (negatively related to blood pressure) and associated pulse wave velocity (PWV) have been extensively used to develop novel cuffless and continuous blood pressure measurements using wearable PPG sensors (Ding and Zhang, 2019). In early works, several PPG pulse wave characteristics have been extracted to determine PTT, including PPG valley (Babchenko et al., 2000; Nitzan et al., 2002; Allen et al., 2008), PPG peak (Zhang and Zhang, 2006; Allen et al., 2008; Wagner et al., 2010; Kortekaas et al., 2012; Li et al., 2014), peak of the first derivative of PPG (Yoon et al., 2009; Kortekaas et al., 2012; Kim et al., 2013), and peak of the second derivative of PPG (Teng and Zhang, 2006; Kortekaas et al., 2012), etc.

PPG measurement is influenced by many factors, including (but not limited to) body site of measurement, breathing pattern, age, etc. All these factors may affect the waveform quality of the PPG data, and subsequently cause challenges in extracting PPG waveform features. Hartmann et al. found that the PPG signals measured from the fingertip achieved the highest percentage of analyzable waveforms for feature extraction among six measurement sites of finger, wrist under, wrist upper, arm, earlobe, and forehead (Hartmann et al., 2019). In addition, the age-related changes of vascular biomechanical properties, e.g., artery stiffness, can significantly influence the PPG waveform and the location of characteristic points (Allen et al., 2020). It has been reported that PWV, the gold standard for evaluating arterial stiffness, was correlated with age in healthy adults (Koivistoinen et al., 2007; Schwartz et al., 2019). Allen et al. observed multiple age-related changes in PPG pulse shape characteristics measured at different body sites, with small but significant overall elongation of the systolic rising edge (Allen and Murray, 2003). They found a significant correlation between aging and PTT shortening (Allen and Murray, 2002).

In addition to the abovementioned physiological factors, preprocessing of raw PPG signals may also incur some changes on PPG waveform features, particularly the timing information. In many wearable applications, raw PPG signals are filtered before feature extraction. Filtering can change the waveform of PPG signals and the timing of feature points (Liu et al., 2021). At present, the finite impulse response (FIR) and infinite impulse response (IIR) filters are widely applied in PPG signal processing. IIR filters offer a number of advantages over other types of filters, such as their ability to achieve a high degree of signal attenuation and their applicability in digital signal processing systems. Whereas, the nonlinear phase response of the IIR filter can deform PPG signals and affect the timing of PPG waveform feature points (Allen and Murray, 2004). In our previous study, we observed that IIR filtering can significantly change the characteristics of PPG waveforms (e.g., peaks and valleys) with the average time shift over 0.1s (Liu et al., 2021). Hence it was noted that filtering parameters should be quoted to support the reproducibility of PPG-related studies (Liu et al., 2021; Charlton et al., 2022). In this paper, we continue this line of thought and research methodology on IIR filtering which will establish the groundwork for future research on FIR and other filters.

Considering the importance of PTT for measuring important cardiovascular signs (e.g., blood pressure, vascular elasticity), it is valuable to study all possible sources leading to PTT measurement errors. So far, little has been reported on how filtering-induced time shift in PPG signal preprocessing affects PTT measurement, which is largely due the lack of a standardized PPG signal processing workflow. The filtering parameters of many commercial wearable PPG sensors are unrevealed. In early studies, the filtering parameters were not uniform and narrow frequency bands were widely used, e.g., 0.5–4 Hz (Wang et al., 2007; Vogel et al., 2009) and 0.8–4 Hz (Poh et al., 2012).

This work took the first step toward quantitatively assessing the effect of PPG pulse filtering on PTT changes. Specially, eight waveform feature points from PPG signals were used to define 8 types of PPT for studying the PPT definition effect, and PPG signals were collected from a wide range of age for studying the possible aging effect.

## 2 Materials and methods

### 2.1 Subjects

A total of one hundred participants (age: 44 ± 14 years, age range: 20–71 years; 48 males and 52 females) were recruited from staff, students and their relatives in Newcastle Hospitals and Newcastle University with written informed consent. The participants were equally distributed in five age groups (i.e., 20–29, 30–39, 40–49, 50–59, and over 60 years old, 20 subjects in each). No participant suffered cardiovascular disease before. This study received ethical permission from the Faculty Research Ethics Panel at Anglia Ruskin University (FMSFREP/17/18205), and all participants provided their written informed consent. The experimental procedures involving human subjects described in this work complied with the principles in the Declaration of Helsinki by World Medical Association in 2000.

### 2.2 Measurement procedure

**FIGURE 1:**
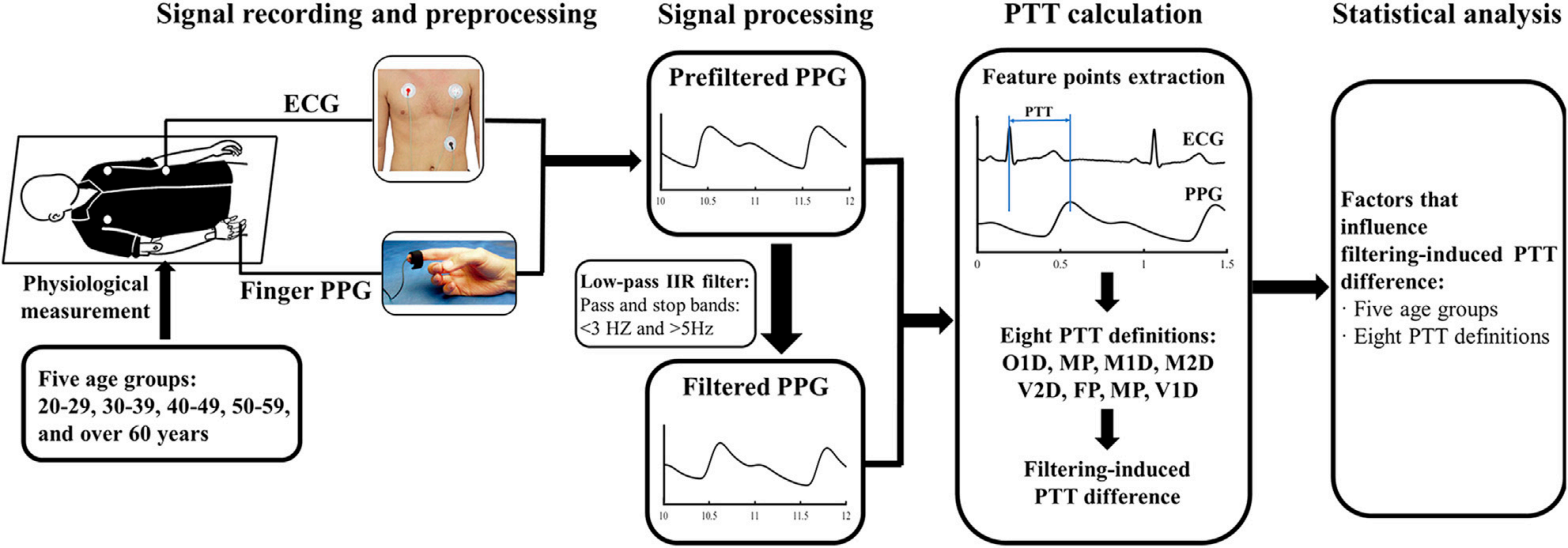
Schematic diagram of the measurement system.

Figure 1 illustrates the schematic diagram of the PPG and ECG measurement system. The experiment was performed in a thermostatic room maintaining the temperature at 23°C ± 1°C. In order to stabilize the cardiovascular system, each participant was guided to lie supine on a couch and rest for 5 min. Their arms were placed parallel to the body without any movement. During the experiment, the ECG and PPG signals were recorded, reviewed, and saved using the MP160 data acquisition system with the Biopac AcqKnowledge software. The sensors to measure ECG and PPG signals were attached to thoracic area and right index fingertip, respectively. The participants were informed to keep a normal breathing. When the ECG and finger PPG signals on the monitor screen were stable, they were recorded simultaneously at a sample rate of 2,500 Hz for 120 s. The operator monitored the data during the recording, reviewed the whole data segments after the recording, and then saved the data with adequate quality. If any error or low-quality segment appeared, the data recording was repeated.

### 2.3 Signal preprocessing and filtering

The recorded data were anonymized and imported to MATLAB (R2021b; MathWorks Inc. Natick, United States) for signal processing. The ECG signals were first pre-processed with a 4th-order Butterworth band-pass filter (passband: 0.05–35 Hz, stopbands: <0.01 Hz and >40 Hz) to remove the baseline drifts (i.e., low-frequency noises) and high-frequency noise, followed by a wavelet transformation to further remove the remaining low-frequency noises due to the slant stopband edges. Specially, the Daubechies 8 wavelet (db8) was used for 11-level decomposition. As compared with a band-pass filter, the discrete wavelet transform could perform better in terms of eliminating high-frequency noise (e.g., electrocardiogram noise, power line noise, etc.) while keeping the morphology feature points of the ECG signal (Zhao et al., 2022). The approximation coefficient of the wavelet decomposition at the 11th level, which contained low-frequency drift component, was replaced by zero. Then, the signal was reconstructed based on the new coefficients to obtain the preprocessed ECG signal.

The raw PPG signals were preprocessed with a high-pass infinite impulse response (IIR) filter (1 zero and 10 poles, passband: >0.5 Hz, stopband: <0.2 Hz) to remove the baseline drifts. A low-pass IIR filter (1 zero and 16 poles, passband: <20 Hz, stopband: >30 Hz) was then used to remove the high-frequency noises which included the 50 Hz power line and electrophysiological noises.

**FIGURE 2:**
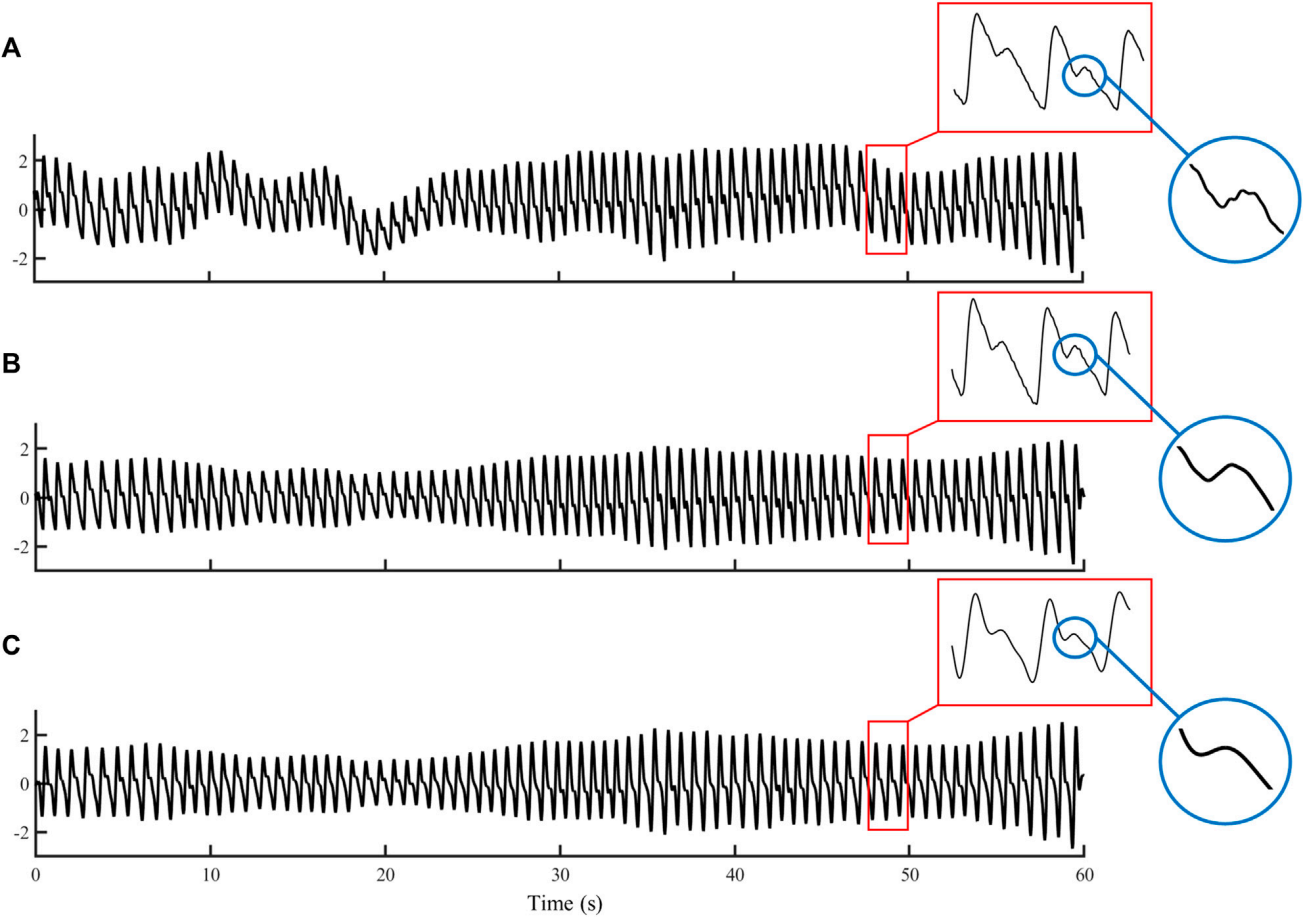
Example waveforms of **(A)** raw PPG, **(B)** preprocessed PPG, and **(C)** filtered PPG signals with a duration of 60 s.

To investigate the effect of filtering on PTT measurement, the preprocessed PPG signals were further filtered with a low-pass IIR filter (1 zero and 13 poles, passband: <3 Hz, stopband: >5 Hz). The details of the PPG signal preprocessing could be found in Figure 2 of (Liu et al., 2021). Figure 2 illustrates the removal of baseline wondering and high-frequency noises (see the enlarged circle) in preprocessing, and further smoothing in the IIR filtering.

### 2.4 Definition of PTT

**FIGURE 3:**
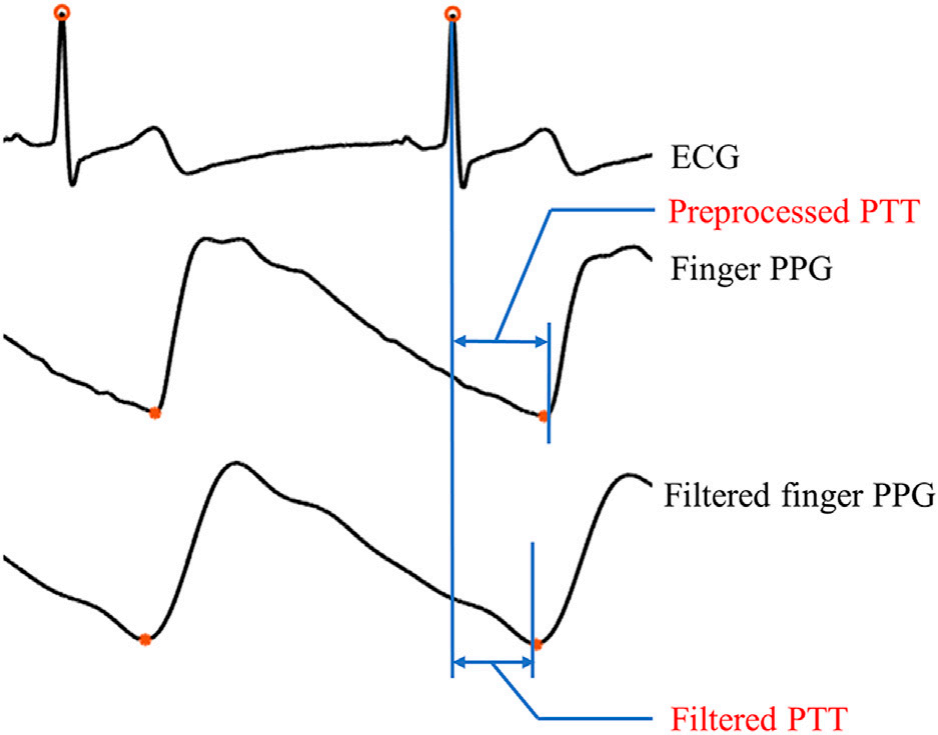
Illustration of two types of PTT, i.e., preprocessed PTT and filtered PTT. Here, the preprocessed PTT refers the interval between the ECG R-wave and the end-of-diastolic valley of the preprocessed PPG signal, while the filtered PTT is the interval between the ECG R-wave and the end-of-diastolic valley of the filtered PPG signal.

PTT is usually defined as the time between the R-peak of the electrocardiogram (ECG) and a reference point on systolic PPG signal segment. The reference point can be derived from different PPG features (e.g., end-of-diastolic valley, systolic peak, see Figure 3), which leads to different PTT definitions.

**FIGURE 4:**
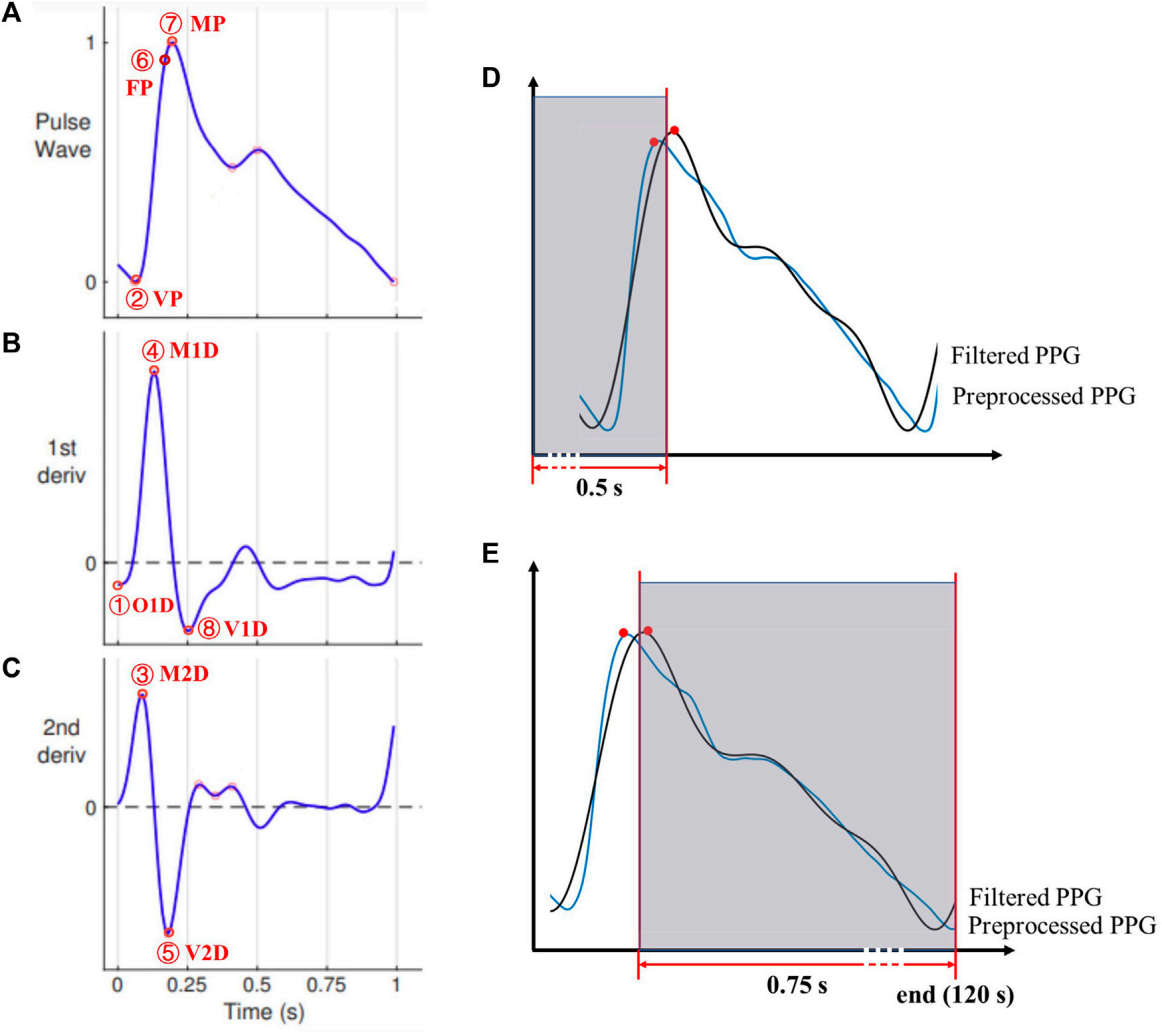
The characteristic points on **(A)** the PPG signal and its **(B)** first and **(C)** second derivatives. Points 1-8 denote O1D, VP, M2D, M1D, V2D, FP, MP, and V1D, respectively. Adopted from (Mejia-Mejia et al., 2022). **(D)** and **(E)**: Corresponding PPG waveform characteristics points of both signals (preprocessed and filtered) were excluded from the analysis if any of them falls in the first 0.5s **(D)** or last 0.75s of the recording **(E)**.

This work selected eight different PPG pulse waveform characteristics to define eight PTTs. The eight PPG pulse waveform characteristics are (see Figure 4).

(1) Onset point of the first derivative (O1D): the onset of the first derivative of PPG in a cardiac cycle.
(2) Valley point of PPG (VP): the point corresponding to the minimum value of the PPG in a cardiac cycle which is located at the end of diastole.
(3) Maximum second derivative (M2D): the point corresponding to the maximum value of the second derivative of PPG in a cardiac cycle.
(4) Maximum first derivative (M1D): the point corresponding to the maximum value of the first derivative of PPG in a cardiac cycle.
(5) Valley point of the second derivative (V2D): the point corresponding to the minimum value of the second derivative of PPG in a cardiac cycle.
(6) Forward peak of PPG (FP): the point that has the maximum value in a cardiac cycle of the forward PPG wave.
(7) Maximum peak of PPG (MP); the systolic peak point that has the maximal PPG value in a cardiac cycle.
(8) Valley point of the first derivative of PPG (V1D): the point corresponding to the minimum value of the first derivative of PPG in a cardiac cycle.

The derivatives were approximated using backward difference calculated from adjacent sampling points. Therefore, the first and second derivatives of the PPG signal started from the second and third sampling points, respectively. Considering the high sampling frequency (i.e., 2,500 Hz), the error caused by the approximation was very limited (< $4\times 10^{-4}$ s for the timing of any characteristic point). The characteristic points were detected from the extrema (i.e., peak and valley points) of PPG and its derivatives, as well as the decomposition of forward and backward pulse waves. The details of defining and detecting characteristic points can be found in our early works (Liu et al., 2021; Lin et al., 2022).

To prevent inaccurate readings at the immediate start and end of a PPG recording, any characteristic point was excluded from the analysis if it or its ‘counterpart’ (i.e., any of the preprocessed or filtered one) fell in the first 0.5 s (e.g., in Figure 4D, the pair of peak points are excluded) or the last 0.75 s (Figure 4E). To exclude the missing or erroneous feature points, any detected feature point was excluded if there was no ‘counterpart’ point within ±0.3 s of the detected feature point. The time axis was unchanged (i.e., no shift of any signal) during signal processing.

As to ECG signals, the R wave peak was detected as the maximal value in a cardiac cycle using the Pan Tompkins method (Sathyapriya et al., 2014). To prevent inaccurate readings at the immediate start and end of an ECG recording, similar as in PPG preprocessing, any R peak point was excluded if it or its counterpart was in the first 0.5 s or the last 0.5 s. When processing the noisy PPG signals in some cardiac cycles, only the valleys within 100–500 ms after the ECG R-peak (i.e., 100 ms≤ PTT ≤500 ms) were selected for analysis. For each PPG signal (preprocessed or filtered), the PTT was calculated as the mean value of PTTs of all included cardiac cycles.

### 2.5 Statistical analysis

For each participant, the filtering-induced PTT difference was calculated between the PTT values derived from the filtered and preprocessed PPG signals. The relative PTT difference was calculated as:

$${RD}_{PTT}=\left({PTT}_{filtered}-{PTT}_{preprocessed}\right)/{PTT}_{preprocessed}$$ (1)

For each subject, the PTT difference and ${RD}_{PTT}$ were averaged in all included cardiac cycles. The ratio between the mean and standard deviation of ${RD}_{PTT}$ in all included cardiac cycles was also calculated to estimate the intra-subject variation of filtering-induced PPT changes. Statistical analysis was performed using SPSS (Version 24.0, IBM Corp.; Armonk, NY, United States) and R programming language, version 4.1.0 (R Core Team, 2021). Considering the data size, Shapiro–Wilk test was performed to investigate the normality of data distribution. Normal distribution was defined as *p* > 0.05 in Shapiro–Wilk test.

To investigate if there was any significant effect of age, PTT definition, or their interaction on the filtering-induced PTT difference or RD_PTT_, the analysis of variance (ANOVA) or Scheirer–Ray–Hare test was performed. ANOVA was performed on normally distributed data where the homogeneity of variance was satisfied (defined as *p* > 0.05 in Levene’s test), otherwise the Scheirer–Ray–Hare test was performed as a substitute.

To further investigate the difference between age groups, or between PTT definitions, and to identify the age group and the PTT definition with the highest reliability (i.e., with the least filtering-induced PTT changes), the *post hoc* analysis was performed, i.e., least significant difference tests and Dunn’s Kruskal–Wallis multiple comparisons for ANOVA and the Scheirer–Ray–Hare test, respectively. In the Dunn’s Kruskal–Wallis multiple comparisons, the *p*-value was adjusted via the Benjamini–Hochberg method (Benjamini and Hochberg, 1995).

Finally, to investigate quantitatively the aging effect on filtering-induced PTT difference and its relative difference, linear regression analysis was performed. The R-squared value was calculated to evaluate the strength of the linear relationship. A significant linear relationship was defined as r > 0.5 (R-squared >0.25) and *p* < 0.05. A strong linear relationship was defined as r > 0.8 (R-squared >0.64) and *p* < 0.05. Regression analysis of the data and curve plotting were performed using Graphpad Prism (version 9.0.0, GraphPad Software, United States).

## 3 Results

### 3.1 Effects of age and PTT definition on filtering-induced PTT differences

The ratio between mean and standard deviation of ${RD}_{PTT}$ in included cardiac cycles was below 20% for all PTTs in 72 subjects, indicating limited intra-subject variability of filtering-induced PTT difference. Therefore, in this study, the data analysis was focused on the mean values of filtering-induced PTT changes and ${RD}_{PTT}$ .

**FIGURE 5:**
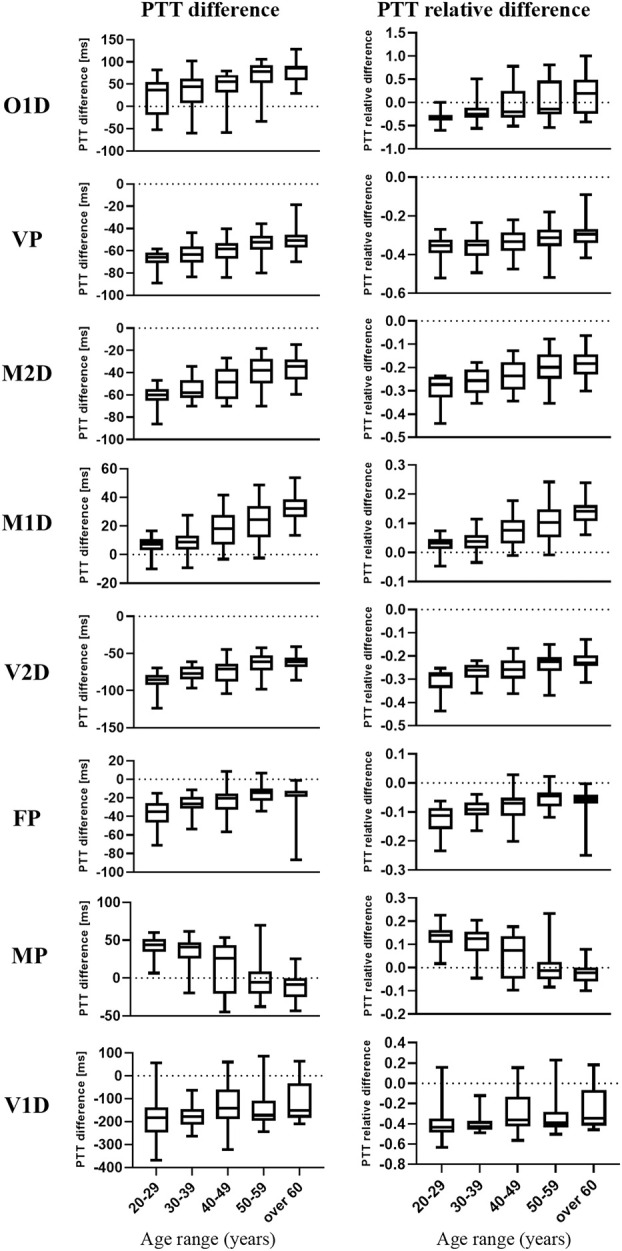
Box and whisker plots of the filtering-induced difference and relative difference of PTT in five age groups and eight PTT definitions. The medians are represented by the horizontal lines within the boxes while the first and third quartiles are represented by the box boundaries.

Figure 5 shows the distribution of filtering-induced PTT difference and its relative difference in five different age ranges and eight PTT definitions. The distribution of filtering-induced PTT difference and relative difference did not satisfy the homogeneity of variance (*p* < 0.05 in Levene’s test for both). Therefore, the Scheirer-Ray-Hare test was performed. As shown in Tables 1, 2, there were significant effects of age and PTT definition (*p* < 0.001 for both) on filtering-induced PTT difference and its relative difference, whereas, the effect of interaction between age and PTT definition is insignificant.

**TABLE 1:** Results of Scheirer-Ray-Hare test regarding the PTT difference. Asterisk denotes significant difference.

	H	*p*-value
Age	22.29	<0.001*
PTT Definition	530.37	<0.001*
Age & PTT Definition Interaction	35.89	0.146

**TABLE 2:** Results of Scheirer-Ray-Hare test regarding the PTT relative difference. Asterisk denotes significant difference.

	H	*p*-value
Age	23.54	<0.001*
PTT Definition	484.48	<0.001*
Age & PTT Definition Interaction	36.87	0.122

### 3.2 Comparison between age groups and PTT definitions

As shown in Figure 5, in most PTT definitions, there is a consecutive and monotonic trend in filtering-induced PTT difference across different age groups (e.g., increase and decrease with age for M1D in MP, respectively).

**FIGURE 6:**
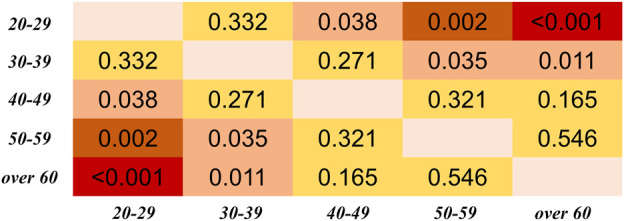
Results of Dunn’s Kruskal–Wallis multiple comparisons between the five age groups regarding the PTT difference, derived from all PTT definitions. The brightness indicates the statistical significance (i.e., higher significance in red color).

**FIGURE 7:**
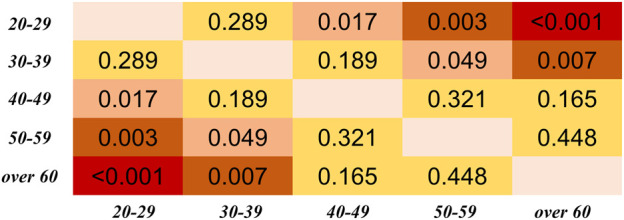
Results of Dunn’s Kruskal–Wallis multiple comparisons between the five age groups regarding the PTT relative difference (RD_PTT_). The brightness indicates the statistical significance (i.e., higher significance in red color).

In Figures 6, 7, in can be observed that the differences in PTT difference and RD_PTT_ both increase with the gap between age groups, where the difference between the youngest and oldest groups (20–29 and >60 years) is the most significant (*p* < 0.001 for both PTT difference and relative difference). Considering all PTT definitions, the age groups (20–29) and (50–59) had the maximum and minimum filtering-induced changes, respectively (for both PTT difference and relative difference, in mean value). Of note, the age groups with maximal and minimal filtering-induced PTT changes were actually definition-specific (Figure 5).

**FIGURE 8:**
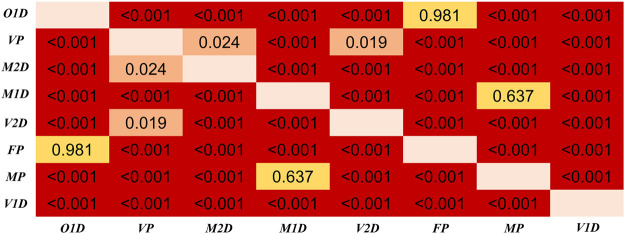
Results of Dunn’s Kruskal–Wallis multiple comparisons between the eight types of PTT definition regarding the PTT difference. The brightness indicates the statistical significance (i.e., higher significance in red color).

**FIGURE 9:**
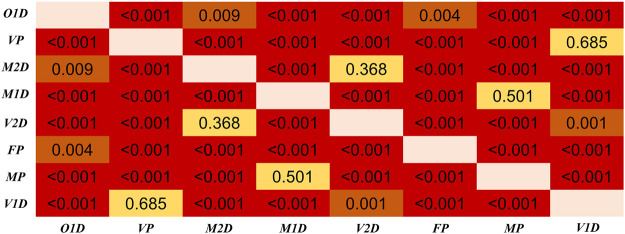
Results of Dunn’s Kruskal–Wallis multiple comparisons between the eight types of PTT definition regarding the PTT relative difference (RD_PTT_). The brightness indicates the statistical significance (i.e., higher significance in red color).

Regarding the differences among PTT definitions, as shown in Figure 8, significant differences in PTT difference were observed (*p* < 0.05) except between O1D and FP, and between MP and M1D. As shown in Figure 9, significant differences in PTT relative difference were observed (*p* < 0.05) except between V2D and M2D, between V1D and VP, as well as between MP and M1D. Therefore, PTT definition has significant influence on the filtering-induced PTT changes. In all subjects, MP had the minimum filtering-induced changes for both PTT difference and RD_PTT_ (mean in all subjects: 16.16 ms and 5.65%, respectively).

### 3.3 Quantitative analysis: age and filtering-induced PTT difference

**FIGURE 10:**
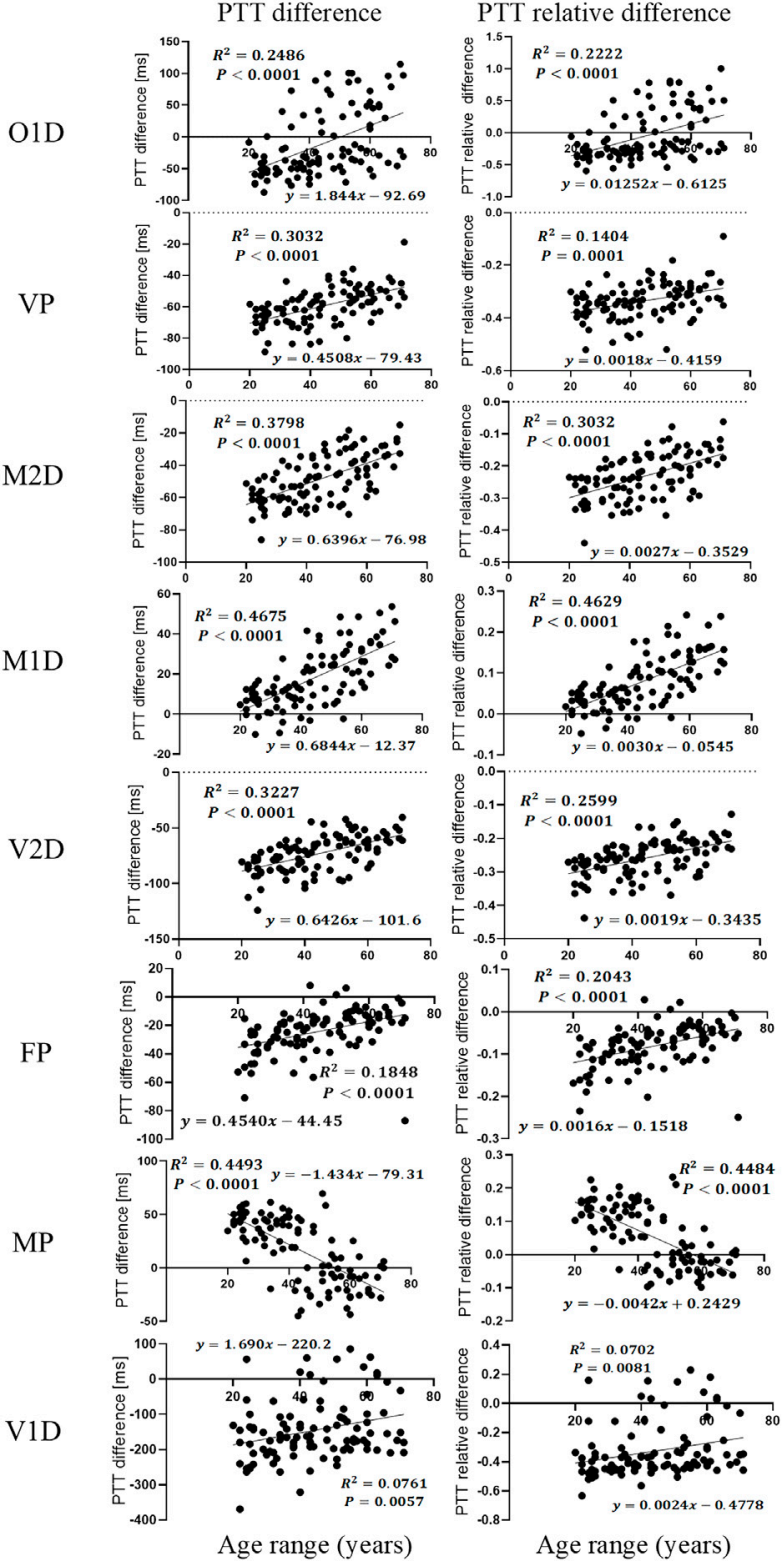
Linear regression plot between age and filtering-induced PTT difference and relative difference (RD_PTT_) under eight definitions of PTT.

As shown in Figure 10, many filtering-induced PTT changes have linear relationships with age, which is in accordance with the trends in Figure 5. The significant linear relationship between age and PTT difference was found in all types of PTT definition group except O1D, FP and V1D. Overall, M1D had the highest strength of the linear relationship ( $r^{2}=0.47$ in PTT difference and $r^{2}=0.46$ in PTT relative difference).

## 4 Discussion and conclusion

The results in this work showed that IIR filtering considerably influenced PTT values by changing the positions of PPG feature points. We observed that filtering-induced PTT changes depended on age and the definition of PTT. In all PTT definitions, the effect of age was consecutive with a monotonous trend. The age group with the least filtering-induced PTT changes depended specifically on PTT definition. Among different PTT definitions, MP showed the highest robustness against the filtering-induced PTT changes. The MP and M1D exhibited the least filtering-sensitive PTT changes, which may explain the lack of significant difference between them in Figures 8, 9. The performance of difference PTTs deserves further investigation on the underlying physiological mechanisms. The linear trends between age and filtering-induced PTT changes indicated the significance of age-based adjustment in PTT estimation. As far as we know, this work is among the first attempts to investigate the filtering-induced PTT changes.

### 4.1 Filtering-induced PTT changes: A neglected concern

PPG signals are typically subject to noises and trends. Therefore, a proper preprocessing plays a key role in many applications, e.g., the functional assessment of autonomic nervous system (Akar et al., 2013). However, at present, there is a lack of comprehensive evaluation of the filtering effect on the accuracy of PTT across different ages. Our results filled this gap and highlighted the importance of age-based adjustment of the filtering-induced inaccuracy in PTT-based applications, e.g., BP estimation.

Currently, despite the increasing diversity in PPG pre-processing techniques, traditional IIR and FIR filers still play an important role because they are easier to design in digital signal processors (Liu et al., 2021; Mejia-Mejia and Kyriacou, 2023). Recently, Mejía-Mejía et al. investigated the effect of PPG filtering strategy in the analysis of pulse rate variability (PRV), and concluded that PRV information can be reliably extracted from PPG signals filtered by elliptic IIR or equiripple or Parks–McClellan FIR filters (Mejia-Mejia and Kyriacou, 2023). We also focused on the IIR filter in this pilot study. Compared with PRV which is measured between consecutive heartbeats, PTT is derived from a much shorter period in a cardiac cycle, and thus can be more sensitive to the filtering-induced time shift of PPG feature points. Our results revealed that the filtering can led to considerable changes in PTT (>39.6%, all age groups in VP), affecting the accuracy in BP estimation. Therefore, filtering-induced PPG waveform deformation deserves further attention in PTT-based BP estimation.

### 4.2 Beyond age: physiological factors that can influence PTT

It is well known that the PTT-BP relationship depends on age (Allen and Murray, 2002; Foo et al., 2005). Allen et al. found a consistent trend in the effect of age on PTT ( $r^{2}=0.48$ ) (Allen and Murray, 2002), while we further observed similar phenomena in the filtering-induced PTT difference (Figures 5, 10). These observations commonly provide the reference for age-adjusted PTT calculation in the future.

Besides age, many other physiological conditions including measurement site, breathing pattern, and neural activities can significantly influence PPG waveform, therefore change the PTT values (Hartmann et al., 2019; Liu et al., 2020; Khalid et al., 2022). The temperature-induced autoregulation can also influence PTT. Teng et al. found that local cold exposure can influence the PTT defined by MP with negligible effect on the PTT defined by VP (Zhang and Zhang, 2006). Furthermore, vascular stiffness increases with age, which has a significant effect on PPG signal waveform (Allen and Murray, 2002). A recent study on oscillography-based BP estimation concludes that, age, BP, and arterial stiffness have complex interaction (Pan et al., 2022). The effect of these physiological factors in PPG waveform changes deserves further investigation.

### 4.3 Technological factors in improving the accuracy of PTT estimation

Some technical issues can influence the PTT values. Recently, Chandrasekhar et al. pointed out that the PPG sensor contact pressure might be another factor that influences the reliability of PTT measurement (Chandrasekhar et al., 2020). Teng et al. found that, during the increase of contacting force, the PTT defined by M2D had the largest overall change (from 200.3 ± 20.6 ms to 225.0 ± 19.3 ms) (Teng and Zhang, 2006), whereas the PTT defined by M1D had the minimum 325 changes (from 245.3 ± 20.2 ms to 261.4 ± 14.2 ms) (Teng et al., 2004) which is in accordance with our observations. They also found that the interaction between age and contact force might influence PTT. As the contact force increased to the mean intra-arterial pressure (zero transmural pressure), PTT increased from 155.2 ± 18.5 ms to 164.7 ± 21.6 ms for the group of elderly subjects and from 186.7 ± 21.0 ms to 201.7 ± 19.5 ms for the group of young subjects (Teng and Zhang, 2007). Therefore, more physiological and technical factors, as well as their interactions, need to be considered to improve the accuracy of PTT-based BP estimation. Since MP and M1D exhibited the least filtering-sensitive PTT changes and showed strongest linear relationships, we recommend using M1D in younger subjects and MP in older subjects in calculating PTT to improve its reliability.

### 4.4 PPG signal preprocessing: towards application-specific standardization

PPG technology provides the possibility of low-cost, non-invasive, and continuous BP measurement for different application scenarios. Recent development of learning-based methods has largely improved the accuracy of BP estimation based on single PPG waveform analysis. PTT-based BP estimation is being considered at a secondary place due to the significant impact of arterial stiffness, individual instability and physical condition on the predetermined hypothetical relationship (Agham and Chaskar, 2021). However, due to its accuracy and reliability, PTT-based BP estimation is still the commonest solution in wearable devices compared with other approaches.

Our results revealed a major limitation of PTT-based methods, i.e., the lack of a framework for standardized filtering and quantitative adjustment of the results. At present, there is a lack of standardized practices in PPG signal acquisition and processing (Charlton et al., 2022). In the majority of PPG studies, there are insufficient details of the settings/parameters of the filters. As summarized in our previous work, the filtering parameters are not uniform, where the lower and upper stop frequencies range around 0.005–0.5 Hz, and 5–20 Hz, respectively, with an inconsistency in key properties such as transition bandwidth and ripples (Liu et al., 2021). Considering the diversity of PPG pre-processing methods, and the interaction between technical with physiological factors in the changes of PTT values, we recommend that the preprocessing of PPG signals can be standardized and tailored to meet different application scenarios, where both technical and physiological factors (e.g., filtering parameters, age, measurement site, etc.) can be considered comprehensively and adjusted quantitatively to improve the accuracy of PTT-based BP estimation.

### 4.5 Limitations and future directions

This is a small-scale pilot study, where other physiological factors as abovementioned were not included to avoid confounding the results. Another major limitation of the study is that it did not include the subjects with very low and high ages (<20 and >70 years). Existing studies showed that the relationship between age and arterial stiffness is non-linear (Vlachopoulos et al., 2011; Laurent et al., 2019). Therefore, the results might not reflect the filtering-induced PTT changes in elderly people who are more liable to hypertension. Also, we only included healthy subjects, without considering the effect of pathological changes on PPG signal waveform. Existing evidence showed that PTT was mildly elevated in patients with heart failure compared with healthy subjects (468 ± 12 vs. 430 ± 23 ms, *p* = 0.001) (Wagner et al., 2010).

In future studies, large-scale, multi-center datasets covering a wider range of age and more pathophysiological conditions could be used to improve the accuracy of PTT calculation and enable a fine-grained PTT estimation framework. More filtering metrics and techniques can be explored to develop a panoramic, standardizable PPG preprocessing framework with high robustness against filtering-induced PPG waveform deformation.

In conclusion, the results in this work showed that the filtering-induced PTT difference was significantly different among different types of PTT definite or among different age groups. The physiological factor including age and PTT definition should be considered in PTT-based application using wearable sensors, e.g., blood pressure estimation.

## Data Availability

The raw data supporting the conclusion of this article will be made available by the authors, without undue reservation.

## References

[B1] Agham N. D. Chaskar U. M. (2021). Learning and non-learning algorithms for cuffless blood pressure measurement: A review. Med. Biol. Eng. Comput. 59 (6), 1201–1222. 10.1007/s11517-021-02362-6 34085135

[B2] Akar S. A. Kara S. Latifoglu F. Bilgic V. (2013). Spectral analysis of photoplethysmographic signals: The importance of preprocessing. Biomed. Signal Process. Control 8 (1), 16–22. 10.1016/j.bspc.2012.04.002

[B3] Allen J. Murray A. (2002). Age-related changes in peripheral pulse timing characteristics at the ears, fingers and toes. J. Hum. Hypertens. 16 (10), 711–717. 10.1038/sj.jhh.1001478 12420195

[B4] Allen J. Murray A. (2003). Age-related changes in the characteristics of the photoplethysmographic pulse shape at various body sites. Physiol. Meas. 24 (2), 297–307. 10.1088/0967-3334/24/2/306 12812416

[B5] Allen J. Murray A. (2004). “Effects of filtering on multisite photoplethysmography pulse waveform characteristics,” in Computers in Cardiology, 2004: IEEE, Chicago, IL, USA, 19-22 September 2004, 485–488.

[B6] Allen J. O’Sullivan J. Stansby G. Murray A. (2020). Age-related changes in pulse risetime measured by multi-site photoplethysmography. Physiol. Meas. 41 (7), 074001. 10.1088/1361-6579/ab9b67 32784270

[B7] Allen J. Overbeck K. Nath A. F. Murray A. Stansby G. (2008). A prospective comparison of bilateral photoplethysmography versus the ankle-brachial pressure index for detecting and quantifying lower limb peripheral arterial disease. J. Vasc. Surg. 47 (4), 794–802. 10.1016/j.jvs.2007.11.057 18381141

[B8] Allen J. (2007). Photoplethysmography and its application in clinical physiological measurement. Physiol. Meas. 28 (3), R1–R39. 10.1088/0967-3334/28/3/R01 17322588

[B9] Babchenko A. Davidson E. Adler D. Ginosar Y. Kurz V. Nitzan M. (2000). Increase in pulse transit time to the foot after epidural anaesthesia treatment. Med. Biol. Eng. Comput. 38 (6), 674–679. 10.1007/bf02344874 11217886

[B10] Benjamini Y. Hochberg Y. (1995). Controlling the false discovery rate: A practical and powerful approach to multiple testing. J. R. Stat. Soc. Ser. B Methodol. 57 (1), 289–300. 10.1111/j.2517-6161.1995.tb02031.x

[B11] Chandrasekhar A. Yavarimanesh M. Natarajan K. Hahn J. O. Mukkamala R. (2020). PPG sensor contact pressure should Be taken into account for cuff-less blood pressure measurement. Ieee Trans. Biomed. Eng. 67 (11), 3134–3140. 10.1109/tbme.2020.2976989 32142414 PMC8856571

[B12] Charlton P. H. Pilt K. Kyriacou P. A. (2022). Establishing best practices in photoplethysmography signal acquisition and processing. Physiol. Meas. 43 (5), 050301. 10.1088/1361-6579/ac6cc4 PMC9136485 35508148

[B13] Ding X. Zhang Y.-T. (2019). Pulse transit time technique for cuffless unobtrusive blood pressure measurement: From theory to algorithm. Biomed. Eng. Lett. 9 (1), 37–52. 10.1007/s13534-019-00096-x 30956879 PMC6431352

[B14] Foo J. Y. A. Wilson S. J. Williams G. Harris M. A. Cooper D. (2005). Age-related factors that confound peripheral pulse timing characteristics in Caucasian children. J. Hum. Hypertens. 19 (6), 463–466. 10.1038/sj.jhh.1001846 15729376

[B15] Hartmann V. Liu H. P. Chen F. Qiu Q. Hughes S. Zheng D. C. (2019). Quantitative comparison of photoplethysmographic waveform characteristics: Effect of measurement site. Front. Physiology 10, 198. 10.3389/fphys.2019.00198 PMC6412091 30890959

[B16] Khalid S. G. Ali S. M. Liu H. P. Qurashi A. G. Ali U. (2022). Photoplethysmography temporal marker-based machine learning classifier for anesthesia drug detection. Med. Biol. Eng. Comput. 60 (11), 3057–3068. 10.1007/s11517-022-02658-1 36063352 PMC9537122

[B17] Khalid S. G. Liu H. Zia T. Zhang J. Chen F. Zheng D. (2020). Cuffless blood pressure estimation using single channel photoplethysmography: A two-step method. IEEE Access 8, 58146–58154. 10.1109/access.2020.2981903

[B18] Kim S. H. Song J. G. Park J. H. Kim J. W. Park Y. S. Hwang G. S. (2013). Beat-to-Beat tracking of systolic blood pressure using noninvasive pulse transit time during anesthesia induction in hypertensive patients. Anesth. Analgesia 116 (1), 94–100. 10.1213/ANE.0b013e318270a6d9 23223109

[B19] Koivistoinen T. Kööbi T. Jula A. Hutri-Kähönen N. Raitakari O. T. Majahalme S. (2007). Pulse wave velocity reference values in healthy adults aged 26–75 years. Clin. Physiology Funct. Imaging 27 (3), 191–196. 10.1111/j.1475-097X.2007.00734.x 17445071

[B20] Kortekaas M. C. Niehof S. P. van Velzen M. H. N. Galvin E. M. Huygen F. Stolker R. J. (2012). Pulse transit time as a quick predictor of a successful axillary brachial plexus block. Acta Anaesthesiol. Scand. 56 (10), 1228–1233. 10.1111/j.1399-6576.2012.02746.x 22845715

[B21] Laurent S. Boutouyrie P. Cunha P. G. Lacolley P. Nilsson P. M. (2019). Concept of extremes in vascular aging. Hypertension 74 (2), 218–228. 10.1161/hypertensionaha.119.12655 31203728

[B22] Li Y. J. Wang Z. L. Zhang L. Yang X. L. Song J. Z. (2014). Characters available in photoplethysmogram for blood pressure estimation: Beyond the pulse transit time. Australas. Phys. Eng. Sci. Med. 37 (2), 367–376. 10.1007/s13246-014-0269-6 24722801

[B23] Lin W. H. Zheng D. C. Li G. L. Zhou H. Chen F. (2022). Investigation on pulse wave forward peak detection and its applications in cardiovascular health. Ieee Trans. Biomed. Eng. 69 (2), 700–709. 10.1109/tbme.2021.3103552 34375276

[B24] Liu H. Allen J. Zheng D. Chen F. (2019). Recent development of respiratory rate measurement technologies. Physiol. Meas. 40 (7), 07TR01. 10.1088/1361-6579/ab299e 31195383

[B25] Liu H. P. Allen J. Khalid S. G. Chen F. Zheng D. (2021). Filtering-induced time shifts in photoplethysmography pulse features measured at different body sites: The importance of filter definition and standardization. Physiol. Meas. 42 (7), 074001. 10.1088/1361-6579/ac0a34 34111855

[B26] Liu H. P. Chen F. Hartmann V. Khalid S. G. Hughes S. Zheng D. C. (2020). Comparison of different modulations of photoplethysmography in extracting respiratory rate: From a physiological perspective. Physiol. Meas. 41 (9), 094001. 10.1088/1361-6579/abaaf0 32731213

[B27] Mejia-Mejia E. Allen J. Budidha K. El-Hajj C. Kyriacou P. A. Charlton P. H. (2022). Photoplethysmography signal processing and synthesis. Photoplethysmography 2022, 69–146. Elsevier. 10.1016/B978-0-12-823374-0.00015-3

[B28] Mejia-Mejia E. Kyriacou P. A. (2023). Effects of noise and filtering strategies on the extraction of pulse rate variability from photoplethysmograms. Biomed. Signal Process. Control 80, 104291. 10.1016/j.bspc.2022.104291

[B29] Nitzan M. Khanokh B. Slovik Y. (2002). The difference in pulse transit time to the toe and finger measured by photoplethysmography. Physiol. Meas. 23 (1), 85–93. 10.1088/0967-3334/23/1/308 11876244

[B30] Pan F. He P. Qian Y. Gao H. Chen F. Liu H. (2022). Changes of oscillogram envelope maximum with blood pressure and aging: A quantitative observation. Physiol. Meas. 43 (11), 115008. 10.1088/1361-6579/aca26d 36374012

[B31] Poh M. Z. Kim K. Goessling A. Swenson N. Picard R. (2012). Cardiovascular monitoring using earphones and a mobile device. Ieee Pervasive Comput. 11 (4), 18–26. 10.1109/mprv.2010.91

[B44] R Core Team (2021). R: A language and environment for statistical computing. Vienna, Austria: R Foundation for Statistical Computing. Available at: https://www.R-project.org

[B32] Sathyapriya L. Murali L. Manigandan T. (2014). “Analysis and detection R-peak detection using modified pan-tompkins algorithm,” in International Conference on Advanced Communication Control and Computing Technologies, Ramanathapuram, India, 08-10 May 2014, 483–487. NEW YORK: Ieee.

[B33] Schwartz J. E. Feig P. U. Izzo J. L. (2019). Pulse wave velocities derived from cuff ambulatory pulse wave analysis. Hypertension 74 (1), 111–116. 10.1161/HYPERTENSIONAHA.119.12756 31132952 PMC6679929

[B43] Teng X. F. Poon C. C. Zhang C. Zhang Y. T. (2004). “Study on the effect of contacting force on pulse transit time,” in2004 2nd IEEE/EMBS International Summer School on Medical Devices and Biosensors, Hong Kong, China, June 26, 2004–July 02, 2004 IEEE, 111–114. 10.1109/ISSMD.2004.1689575

[B34] Teng X. F. Zhang Y. T. (2006). The effect of applied sensor contact force on pulse transit time. Physiol. Meas. 27 (8), 675–684. 10.1088/0967-3334/27/8/002 16772666

[B35] Teng X. F. Zhang Y. T. (2007). Theoretical study on the effect of sensor contact force on pulse transit time. Ieee Trans. Biomed. Eng. 54 (8), 1490–1498. 10.1109/tbme.2007.900815 17694870

[B36] Vlachopoulos C. Terentes-Printzios D. Stefanadis C. (2011). When the arteries get tough, the tougher do not get going. Hypertens. Res. 34 (7), 793–794. 10.1038/hr.2011.49 21525948

[B37] Vogel S. Hulsbusch M. Hennig T. Blazek V. Leonhardt S. (2009). In-ear vital signs monitoring using a novel microoptic reflective sensor. Ieee Trans. Inf. Technol. Biomed. 13 (6), 882–889. 10.1109/titb.2009.2033268 19846385

[B38] Wagner D. R. Roesch N. Harpes P. Kortke H. Plumer P. Saberin A. (2010). Relationship between pulse transit time and blood pressure is impaired in patients with chronic heart failure. Clin. Res. Cardiol. 99 (10), 657–664. 10.1007/s00392-010-0168-0 20473677

[B39] Wang L. Lo B. P. L. Yang G. Z. (2007). Multichannel reflective PPG earpiece sensor with passive motion cancellation. Ieee Trans. Biomed. Circuits Syst. 1 (4), 235–241. 10.1109/tbcas.2007.910900 23852004

[B40] Yoon Y. Cho J. H. Yoon G. (2009). Non-constrained blood pressure monitoring using ECG and PPG for personal healthcare. J. Med. Syst. 33 (4), 261–266. 10.1007/s10916-008-9186-0 19697692

[B41] Zhang X. Y. Zhang Y. T. (2006). The effect of local mild cold exposure on pulse transit time. Physiol. Meas. 27 (7), 649–660. 10.1088/0967-3334/27/7/008 16705262

[B42] Zhao X. Y. Zhang J. C. Gong Y. L. Xu L. H. Liu H. P. Wei S. J. (2022). Reliable detection of myocardial ischemia using machine learning based on temporal-spatial characteristics of electrocardiogram and vectorcardiogram. Front. Physiology 13, 854191. 10.3389/fphys.2022.854191 PMC9192098 35707012

